# Role of pelitinib in the regulation of migration and invasion of hepatocellular carcinoma cells via inhibition of Twist1

**DOI:** 10.1186/s12885-023-11217-2

**Published:** 2023-07-27

**Authors:** Sewoong Lee, Eunjeong Kang, Unju Lee, Sayeon Cho

**Affiliations:** Laboratory of Molecular and Pharmacological Cell Biology, College of Pharmacy, Chung-Ang University, Seoul, 06974 Republic of Korea

**Keywords:** Twist1, Epithelial-mesenchymal transition, Pelitinib, Akt, MAPK, HCC

## Abstract

**Background:**

Overexpression of Twist1, one of the epithelial-mesenchymal transition-transcription factors (EMT-TFs), is associated with hepatocellular carcinoma (HCC) metastasis. Pelitinib is known to be an irreversible epidermal growth factor receptor tyrosine kinase inhibitor that is used in clinical trials for colorectal and lung cancers, but the role of pelitinib in cancer metastasis has not been studied. This study aimed to investigate the anti-migration and anti-invasion activities of pelitinib in HCC cell lines.

**Methods:**

Using three HCC cell lines (Huh7, Hep3B, and SNU449 cells), the effects of pelitinib on cell cytotoxicity, invasion, and migration were determined by cell viability, wound healing, transwell invasion, and spheroid invasion assays. The activities of MMP-2 and -9 were examined through gelatin zymography. Through immunoblotting analyses, the expression levels of EMT-TFs (Snail1, Twist1, and ZEB1) and EMT-related signaling pathways such as mitogen-activated protein kinases (MAPKs) and Akt signaling pathways were measured. The activity and expression levels of target genes were analyzed by reporter assay, RT-PCR, quantitative RT-PCR, and immunoblotting analysis. Statistical analysis was performed using one-way ANOVA with Dunnett's Multiple comparison tests in Prism 3.0 to assess differences between experimental conditions.

**Results:**

In this study, pelitinib treatment significantly inhibited wound closure in various HCC cell lines, including Huh7, Hep3B, and SNU449. Additionally, pelitinib was found to inhibit multicellular cancer spheroid invasion and metalloprotease activities in Huh7 cells. Further investigation revealed that pelitinib treatment inhibited the migration and invasion of Huh7 cells by inducing Twist1 degradation through the inhibition of MAPK and Akt signaling pathways. We also confirmed that the inhibition of cell motility by Twist1 siRNA was similar to that observed in pelitinib-treated group. Furthermore, pelitinib treatment regulated the expression of target genes associated with EMT, as demonstrated by the upregulation of E-cadherin and downregulation of N-cadherin.

**Conclusion:**

Based on our novel finding of pelitinib from the perspective of EMT, pelitinib has the ability to inhibit EMT activity of HCC cells via inhibition of Twist1, and this may be the potential mechanism of pelitinib on the suppression of migration and invasion of HCC cells. Therefore, pelitinib could be developed as a potential anti-cancer drug for HCC.

**Supplementary Information:**

The online version contains supplementary material available at 10.1186/s12885-023-11217-2.

## Introduction

Liver cancer, particularly hepatocellular carcinoma (HCC), has seen a surge in incidence over the past few decades [1], with HCC accounting for over 90% of liver cancer cases and carrying a high risk of metastasis [2]. HCC is now ranked third in global mortality rates, and it is typically caused by factors such as infection of hepatitis B and C viruses, cirrhosis, diabetes, and chronic alcohol use [3, 4]. Although treatments such as liver resection, chemotherapy, and transplantation are available for HCC patients [3, 5], the overall prognosis for metastatic HCC remains poor due to the high rates of recurrence and metastasis after surgery [6]. As such, there is a growing need for new therapeutic strategies to prevent the metastasis of HCC. Currently, neoadjuvant therapy has garnered significant interest as a potential treatment for HCC. Several studies have demonstrated that the recent Food and Drug Administration (FDA) approval of the combination of nivolumab and ipilimumab could be administered in the neoadjuvant setting for HCC prior to resection [7, 8]; however, further clinical trials are needed to confirm its efficacy and safety. In addition to neoadjuvant therapy, bioinformatics approaches have been used to predict drug responses and identify potential therapeutic targets for HCC. Several studies have identified the target genes of drug in HCC through bioinformatics screening [9–11]. In addition, organ-on-a-chip technologies have been used for in vitro drug response and sensitivity testing in HCC, with FDA-approved drugs such as sorafenib, lenvatinib, and regorafenib showing promising results [12–14]. However, despite these advances, there is still a need to develop new and effective therapeutic strategies for the treatment of HCC.

The process of metastasis consists of a series of sequential steps, including infiltration of the cancer cells into the adjacent tissue, migration via intravasation, extravasation, and proliferation in metastatic sites [15]. Cancer cells, when they invade into adjacent cell layers, undergo epithelial-mesenchymal transition (EMT) [16, 17]. EMT is a process of trans-differentiation where epithelial cells largely lose their epithelial characteristics and take on properties typical of mesenchymal cells [18]. During EMT, a family of cadherin proteins plays a pivotal role in cell-to-cell adhesion [19]. Its molecular hallmark is the loss of epithelial markers such as E-cadherin, accompanied by the upregulation of mesenchymal markers such as N-cadherin. In addition, EMT induces the upregulation of proteases such as matrix metalloproteinase (MMP)-2 or -9 to degrade extracellular matrix (ECM) [20]. These changes confer metastatic properties on cancer cells by enhancing motility and invasion.

The epithelial and mesenchymal markers are generally regulated by the EMT-transcription factors (EMT-TFs) Twist1/2, Snail1/2/3, and ZEB1/2 [21]. These EMT-TFs are activated through several signaling pathways, including Akt and mitogen-activated protein kinases (MAPK) consisting of extracellular signal-regulated kinase (ERK), c-Jun N-terminal kinase (JNK), and p38 [22, 23]. These signaling pathways are the most studied intracellular signaling pathways associated with EMT-TFs [24–27]. Twist1 is a highly conserved member of the basic helix-loop-helix proteins and is responsible for the transcriptional regulation of mesenchymal cell lineages [21]. Several studies have demonstrated that the overexpression of Twist1 is upregulated in several cancer types including breast, liver, prostate, melanomas, and endometrial cancer [28–31]. A study by Sun et al. has shown that Twist1 expression is correlated with vascular formation in HCC [32]. Additionally, Twist1 is associated with cancer cell invasion and poor survival in HCC patients [33, 34]. As Twist1 is critical in promoting EMT and metastasis in HCC cells, the identification of a novel compound targeting Twist1 and its underlying mechanism of action could provide valuable insights into the development of improved therapeutic strategies for HCC.

Pelitinib is a well-known inhibitor of epidermal growth factor receptor tyrosine kinase (EGFR-TK) [35]. It interacts with the ATP-binding cassette superfamily G member 2 transporters with great affinity, resulting in the elimination of lung cancer stem cell-like cells [35]. Several studies have shown that pelitinib targets human epidermal growth factor receptor 2 and ERK [36, 37]. MAPK and Akt signaling pathways are two significant signaling pathways downstream of EGFR [38]. Hyper-activation of EGFR signaling has been detected in several cancers, including colon, head and neck, ovarian, and breast cancers [39]. Pelitinib was evaluated in a phase 2 clinical trial for the treatment of colorectal and non-small cell lung cancer (NSCLC), but further trials have been suspended [35, 40]. Although a study by Kim et al. has shown that pelitinib inhibits the proliferation of HCC cells [37], studies from the perspective of EMT-TFs for HCC have not yet been conducted.

In this study, we investigated the anti-migration and anti-invasion effects of pelitinib as well as the underlying molecular mechanisms in various HCC cell lines, including Huh7, Hep3B, and SNU449. Our data suggested that these inhibitory effects of pelitinib in HCC might involve the inhibition of Akt and MAPK signaling pathways, followed by suppression of Twist1.

## Methods

### Cell culture

Huh7, Hep3B, and SNU449 cells were purchased from the Korean Cell Line Bank. Huh7 and SNU449 cells were grown in Roswell Park Memorial Institute 1640 medium. Hep3B cells were grown in Dulbecco’s modified Eagle’s medium. Both media contained 10% fetal bovine serum (FBS; WELGENE Inc., Gyeongsan, Korea) and 1% penicillin/streptomycin (Thermo Fisher Scientific Inc., MA, USA). The cells were incubated at 37 °C in a humidified atmosphere with 5% CO_2_.

### Antibodies and reagents

Mouse monoclonal anti-phospho (p)-Akt1/2/3 (Ser473/474/472; cat. No. sc-514032), mouse monoclonal anti-Akt1/2/3 (cat. No. sc-81434), mouse monoclonal anti-JNK (cat. No. sc-7345), mouse monoclonal anti-β-actin (cat. No. sc-47778), mouse monoclonal anti-p38 (cat. No. sc-7972), mouse monoclonal anti-Twist1 (cat. No. sc-81417), mouse monoclonal anti-Snail1 (cat. No. sc-393172), and mouse monoclonal anti-ZEB1 (cat. No. sc-515797) were purchased from Santa Cruz Biotechnology, Inc. (Texas, USA). Rabbit polyclonal anti-p-p38 (Thr180/Tyr182; cat. No. #9211), mouse monoclonal anti-p-ERK1/2 (Thr202/Tyr204; cat. No. #9106), rabbit polyclonal anti-p-JNK1/2 (Thr183/Tyr185; cat. No. #4668), and rabbit polyclonal anti-ERK1/2 (cat. No. #9102) were purchased from Cell Signaling Technology, Inc. (MA, USA). Mouse monoclonal anti-N-cadherin (cat. No. 610720) and mouse monoclonal anti-E-cadherin (cat. No. 610182) antibodies were purchased from BD Biosciences (CA, USA). Glyceraldehyde 3-phosphate dehydrogenase (GAPDH) antibody (GTX100118) was purchased from GeneTex (CA, USA). Primary antibodies were diluted at 1:1000 in 5% of bovine serum albumin (BSA). Polyclonal anti-rabbit IgG-HRP (cat. No. LF-SA8002) and polyclonal anti-mouse IgG Fc-HRP (cat. No. LF-SA8001) were purchased from AbFrontier (Seoul, Korea) and diluted at 1:5000 in 5% of skim milk. LY294002 (440202–5 MG) was purchased from Calbiochem (CA, USA). U0126 (U120-1 MG) and SP600125 (S5567-10 MG) were purchased from Sigma-Aldrich (MO, USA). SB203580 (S1076-25 MG) was purchased from Selleckchem (TX, USA).

### Cell viability assay

Huh7, Hep3B, and SNU449 cells (4.5 × 10^4^ cells/well) seeded on 96-well plates were treated with various concentrations of pelitinib (0.2, 0.5, 1, and 2 μM) at 37 °C for 48 h. The cytotoxic effect was measured using the EZ-Cytox kit (Daeil Lab, Seoul, Korea). The cells were incubated with EZ-Cytox cell viability assay solution (diluted 1:20 in culture medium) at 37 °C for 1 h. The absorbance at 450 nm (A_450_) and reference absorbance at 650 nm (A_650_) were measured on a Synergy H1 Microplate Reader (BioTek Instruments, Inc., VT, USA), and the cell viability was calculated as the difference A_450_ – A_650_.

### Wound healing assay

Cells were seeded on 6-well plates at a density of 1.2 × 10^6^ cells/well and incubated overnight. A straight scratch was introduced using a 0.5 mm scratcher (SPL Life Sciences, Gyeonggi-do, Korea) on a confluent monolayer of cells. Subsequently, the cells were treated with various concentrations of pelitinib in culture media supplemented with 1% FBS. The cells were observed at the indicated times (0 h, 24 h, and 48 h) with a JuLi stage real-time history recorder (NanoEnTek Inc., Seoul, Korea). The wound closure was calculated as the ratio of the wound area at 24 h or 48 h, to the initial wound size at 0 h. The results were visualized on bar graphs. All experiments were performed in triplicates.

### Transwell invasion assay

Transwell plates (24-well plates with 8 μm of pore size; SPL Life Sciences, Gyeonggi-do, Korea) were coated with 0.5 mg/ml of Matrigel solution (BD Biosciences, CA, USA) and incubated at 37 ℃ for 2 h. In the upper chamber, 2 × 10^5^ cells/well were seeded in 250 µl of culture medium supplemented with 1% FBS in the absence or presence of pelitinib. The lower chamber was then filled with 500 µl of culture media containing 10% FBS. After incubation at 37 ℃ for 21 h, the membranes were fixed with 4% paraformaldehyde at room temperature (RT) for 10 min. Subsequently, the transwell inserts were washed with phosphate-buffered saline (PBS). The transwell membrane was stained with 0.5% crystal violet and incubated at RT for 10 min. The transwell inserts were washed once more with PBS. The cells in the upper chamber were removed with a cotton swab. After the transwell inserts were air-dried, images of random areas of the membrane were taken at 100 × magnification. To calculate the number of invasive cells, the total area covered by stained cells in each image was calculated using Image J software (available from: https://imagej.nih.gov/ij/download.html) and divided by that in the control group. The data was visualized on a bar graph.

### Gelatin zymography

Huh7 cells were seeded at a density of 2 × 10^5^ cells/well in 12-well plates. The cells were treated with serum-free media containing various concentrations of pelitinib for 48 h. After the incubation, the media were mixed with 5 × sodium dodecyl sulfate (SDS)-polyacrylamide sample buffer containing 12 mM trisaminomethane hydrochloride (Tris–HCl) (pH 6.8), 5% glycerol, 0.4% SDS, and 0.02% bromophenol blue. The prepared samples were analyzed on 10% SDS-PAGE gel containing 0.1% gelatin. After electrophoresis, the SDS-PAGE gel was washed with wash buffer containing 50 mM Tris–HCl (pH 7.5), 5 mM CaCl_2_, 1 μM ZnCl, and 2.5% Triton X-100 at RT for 30 min. The gel was incubated in substrate buffer containing 50 mM Tris–HCl (pH 7.5), 1% Triton X-100, 5 mM CaCl_2_, and 1 μM ZnCl at 37 °C for 24 h. After 24 h, the gel was washed three times with distilled water and fixed with fixing buffer (40% methanol and 10% acetic acid) at RT for 30 min. Subsequently, the gel was rinsed with distilled water three times, each time for 10 min. For the visualization of bands, the gel was stained with Coomassie Blue (10% acetic acid, 0.25% Coomassie brilliant blue R-250, and 50% methanol) at RT for 30 min and destained with distilled water at RT overnight.

### Three-dimensional (3D) tumor spheroid invasion assay

A total of 2 × 10^3^ Huh7 cells were seeded in each well of low-attachment 96-well plates and incubated for 3 days. After removing the media, 3 mg/ml of Matrigel diluted in culture media was added. The plate was centrifuged at 1,300 rpm for 3 min at 4 °C and then incubated at 37 ℃ for 18 h. The spheroids were treated with various concentrations of pelitinib in 10% FBS media. All images were taken using a JuLi stage real-time history recorder. The spheroid areas were determined using Image J software. The invaded area was calculated as a ratio of the spheroid size from 0 to 48 h.

### Quantitative reverse transcription polymerase chain reactions (RT-qPCRs)

Huh7 cells were seeded (2 × 10^5^ cells/well) in 12-well plates and treated with various concentrations of pelitinib for 48 h. Total RNA was isolated using Labozol reagent (Cosmogenetech, Seoul, Korea) and 1 μg of RNA was reverse transcribed into complementary DNA (cDNA) using a TOPscript cDNA synthesis kit (Enzynomics, Daejeon, Korea). Quantification of mRNA was performed using a RT-q premix and SYBR Green Q Master (Cosmogenetech, Seoul, Korea) according to the instructions of manufacturer. Quantitative PCR conditions were set as follows: initial denaturation at 95 °C for 5 min followed by between 35–40 cycles of 95 °C for 5 s, annealing at 55 °C for 30 s, and extension at 72 °C for 30 s. GAPDH was used for normalization. Each gene expression level was calculated using the 2^−∆∆∆Cq^ method. The sequences of PCR primers used were listed as follows: *TWIST1* sense, 5’-GGC TCA GCT ACG CCT TCT C-3’; *TWIST1* antisense, 5’-CTC CTT CTC TGG AAA CAA TGA CAT-3’, *GAPDH* sense, 5’-GGT GTG AAC CAT GAG AAG TAT GA-3’; *GAPDH* antisense, 5’-GAG TCC TTC CAC GAT ACC AAA G-3’, *CDH1* sense, 5’-TTC CCA ACT CCT CTC CTG-3’; *CDH1* antisense, 5’-AAA CCT TGC CTT CTT TGT C-3’, *CDH2* sense, 5’-CCT CCA GAG TTT ACT GCC ATG AC-3’; *CDH2* antisense, 5’-GTA GGA TCT CCG CCA CTG ATT C-3’.

### Immunoblotting analysis

Huh7 cells were seeded on 6-well plate and incubated overnight. The cells were treated with different concentrations of pelitinib for 48 h and subsequently washed with cold PBS (pH 7.4). Cells were lysed with lysis buffer (150 mM NaCl, 20 mM Tris–HCl [pH 8.0], 0.5% IGEPAL CA-630 [NP-40], 0.5% Triton X-100, 1 mM ethylenediaminetetraacetic acid, 1% glycerol, 2 mM phenylmethylsulfonyl fluoride, 10 mM sodium fluoride, and 1 mM sodium orthovanadate). The lysates were centrifuged at 13,000 rpm at 4 °C for 20 min. Supernatants were transferred to new Eppendorf tubes. Protein concentration was measured using the Bradford protein assay (Bio-Rad Laboratories, Inc., CA, USA) according to the guidelines of the manufacturer. Prepared cell lysates were mixed with 5 × SDS–polyacrylamide sample buffer containing 1% β-mercaptoethanol and boiled at 100 °C for 5 min. The samples were run on 10% or 12% SDS–polyacrylamide gels and transferred to nitrocellulose membranes (Bio-Rad Laboratories, Inc., CA, USA). After the transfer, the membranes were incubated with 5% nonfat-dried skim milk in 1 × Tris‑buffered saline containing 0.05% Tween 20 (TBST) solution on RT for at least 1 h for blocking. The membranes were washed with 1 × TBST solution three times and incubated with primary antibodies in 5% BSA overnight at 4 °C. Subsequently, the membranes were washed 5 times with 1 × TBST and incubated with secondary antibodies at RT for 2 h. The target proteins were detected using an enhanced chemiluminescence immunoblotting detection reagent (Dynebio, Gyeonggi-do, Seoul). The protein bands were quantified with Image J software.

### Twist1 small interfering RNA (siRNA) transfection

The scrambled negative control and Twist1 siRNA were purchased from Bioneer (Daejeon, Korea). The sequences of siRNA used in this experiment were as follows: scrambled siRNA sense, 5’-CCU ACG CCA CCA AUU UCG U-3’ and antisense, 5’-ACG AAA UUG GUG GCG UAG G-3’; Twist1 siRNA sense, 5’-CUG AAC AGU UGU UUG UGU U-3’ and antisense, 5’-AAC ACA AAC AAC UGU UCA G-3’. For transfection, 100 nM of siRNA was mixed with serum-free medium and Lipofectamine 2000 (Thermo Fisher Scientific Inc., MA, USA) and incubated at RT for 30 min. Subsequently, the mixture was applied to Huh7 cells for transfection.

### Statistical analysis and experimental replicates

All experiments in the present study were performed three times. The results are expressed as the means ± standard deviation (SD). Differences between experimental conditions were assessed using one-way ANOVA with Dunnett’s Multiple comparison tests on Prism 3.0 (GraphPad Software, CA, USA). Values of ^a^*p* < 0.05, ^b^*p* < 0.01, and ^c^*p* < 0.001 were considered statistically significant.

## Results

### Pelitinib inhibits the migration of HCC cells without affecting cell viability

**Fig. 1:**
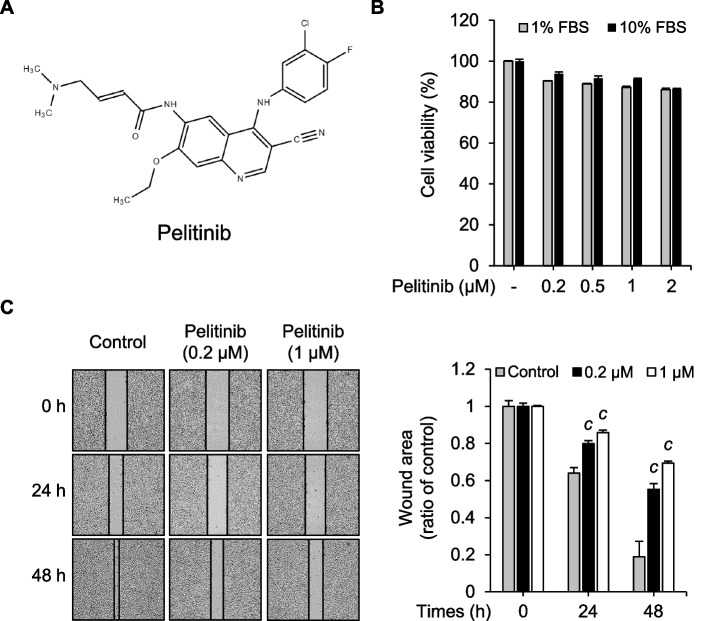
Effects of pelitinib on cell viability and migration in Huh7 cells. **A** The chemical structure of pelitinib. **B** Huh7 cells were exposed to various concentrations of pelitinib for 24 h and 48 h in cell culture media containing 1% or 10% FBS. Using EZ-Cytox kit, the cell viability was assessed. The results were shown as bar graphs in comparison to the pelitinib-untreated control group. **C** The wounds were made by a plastic scratcher in Huh7 cells, and the cells were incubated with various concentrations of pelitinib in 1% FBS-containing media. Microscopic images were captured at indicated time points. The wound area is expressed as the percentage of the initial wound size for each sample and the relative wound closure was displayed as a bar graph. The results displayed are the averages of three independent experiments and values are expressed as means ± SD. The data are analyzed by one-way ANOVA analysis. ^c^*p* < 0.001 relative to the pelitinib-untreated control group

Pelitinib (Fig. 1A) as an EGFR-TK inhibitor has been evaluated in phase 2 clinical trials for colorectal cancer and NSCLC [35, 40], but the molecular mechanisms of its anti-cancer effects from the perspective of EMT on HCC cell lines have not been studied to date. The purpose of this study was to investigate the potential of pelitinib as an anti-metastatic cancer agent in HCC. Prior to subsequent experiments, it was pivotal to evaluate the cytotoxicity of various concentrations of pelitinib in Huh7 cells to find the appropriate concentrations to use in this study. As shown in Fig. 1B, pelitinib applied at concentrations up to 2 μM did not exert significant cytotoxicity on Huh7 cells in culture media with either 1% or 10% FBS. Although the cell viability dropped to approximately 86%, this level of viability is regarded as an indication of non-cytotoxicity [41]. In addition, other HCC cell lines, Hep3B and SNU449, also showed no significant cytotoxicity at various concentrations of pelitinib (Supplement Fig. 1A and B). Wound migration distance in Huh7 cells was measured by wound healing assays to evaluate the ability of pelitinib to suppress cell migration. Pelitinib significantly inhibited the closure of wounds over time (Fig. 1C). To find out whether pelitinib also inhibits the migration of other HCC cell lines, the wound healing assay was performed in Hep3B and SNU449 cells as well. Pelitinib also significantly suppressed the wound closure of these two cell lines in a dose-dependent manner (Supplement Fig. 1C and D). Overall, these data suggest that pelitinib exerts an inhibitory effect on cell migration in HCC cell lines without affecting cell viability.

### Pelitinib inhibits Huh7 cell invasion and the activities of MMP-2 and -9

**Fig. 2:**
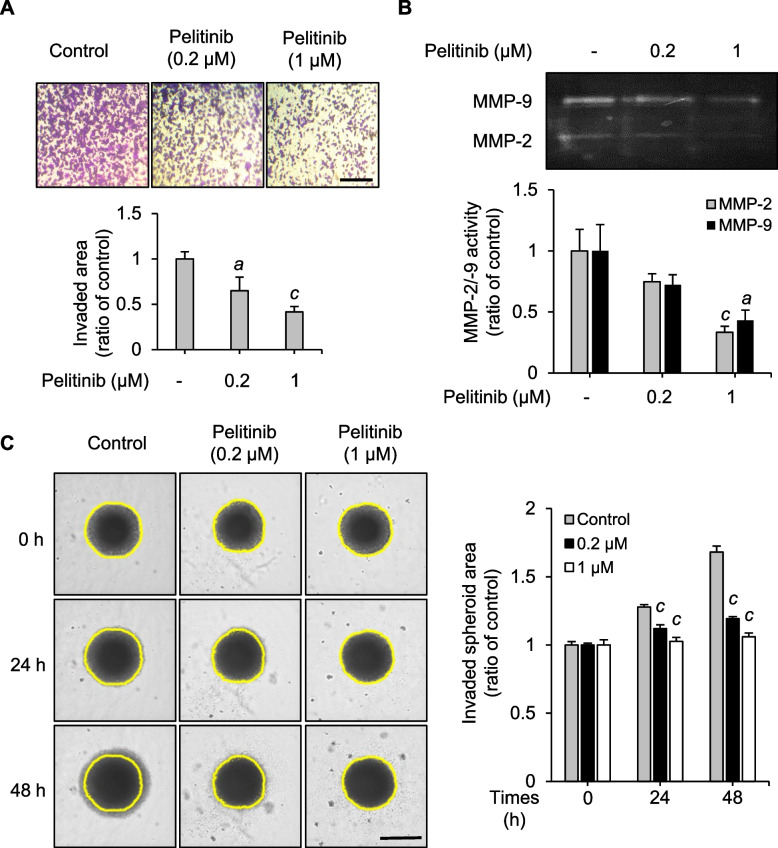
Inhibitory effects of pelitinib on Huh7 cell invasion. **A** Matrigel (0.5 mg/ml) coated on transwell upper chamber for 2 h in 37℃ incubators. Subsequently, Huh7 cells were seeded in the upper chamber and treated with various concentrations of pelitinib in 1% FBS-containing media. In the lower chamber, 10% FBS-containing media were added, and it was incubated at 37 °C for 21 h. After 21 h of incubation, cotton swabs were used to remove cells from the upper chamber, and images were then taken. Scale bar for all images is 250 µm. The relative invaded areas were shown as a bar graph. The results displayed are the averages of three independent experiments and values are expressed as means ± SD. The data are analyzed by one-way ANOVA analysis. ^a^*p* < 0.05 and ^c^*p* < 0.001 relative to the pelitinib-untreated control group. **B** Huh7 cells were treated with various concentrations of pelitinib in serum-free media for 24 h and analyzed by gelatin zymography. SDS-PAGE with 0.1% gelatin was used for the detection of MMP-2 and -9 activities. The bar graph shows quantified band intensities expressed as a ratio of the controls. The results displayed are the averages of three independent experiments and values are expressed as means ± SD. The data are analyzed by one-way ANOVA analysis. ^a^*p* < 0.05 and ^c^*p* < 0.001 relative to the pelitinib-untreated control group. **C** 3D spheroid invasion assay in Huh7 cells treated with different concentrations of pelitinib for 48 h. The microscopic pictures were captured at each time point. Scale bar for all images is 250 µm. The bar graph shows the spheroid area at each time point relative to the initial area (at 0 h) for each sample. In all panels, the data are representative of three independent experiments and expressed as the mean ± SD. The one-way ANOVA is then used to analyze the data. ^c^*p* < 0.001 relative to the pelitinib-untreated control group

As described above, it was confirmed that pelitinib inhibited cell migration in the three HCC cell lines tested. Among these three cell lines, Huh7 cells were chosen for further investigation in this study. The transwell invasion assays were performed using Matrigel to investigate the effect of pelitinib on the invasive behavior of Huh7 cells. As shown in Fig. 2A, pelitinib inhibited the invasive properties of Huh7 cells in a dose-dependent manner. Treatment with 1 μM pelitinib resulted in more than 50% inhibition of invasion compared with the control group. Since MMPs facilitate the degradation and modification of the ECM for metastasis, the activities of MMPs including MMP-2 and -9 are monitored to examine the potential anti-invasion effects of drugs on cancer cells [42]. As shown in Fig. 2B, gelatin zymography was performed to evaluate the activities of MMP-2 and -9 upon pelitinib treatment in Huh7 cells. The activities of MMP-2 and -9 were significantly inhibited by pelitinib. Furthermore, the anti-invasion effect of pelitinib on HCC was confirmed by a 3D culture system mimicking the in vivo cancer microenvironment conditions (Fig. 2C). The invasion of multicellular tumor spheroids into surrounding Matrigel was assessed by monitoring the spheroid area at 0 h, 24 h, and 48 h and examining the changes in spheroid size over time. At the final time point of 48 h, pelitinib-treated group exhibited a smaller spheroid area compared to the untreated control group. These results suggest that pelitinib has the potential to inhibit cancer progression by suppressing cell invasion and the activities of MMP-2 and -9 in Huh7 cells.

### Pelitinib induces the degradation of Twist1

**Fig. 3:**
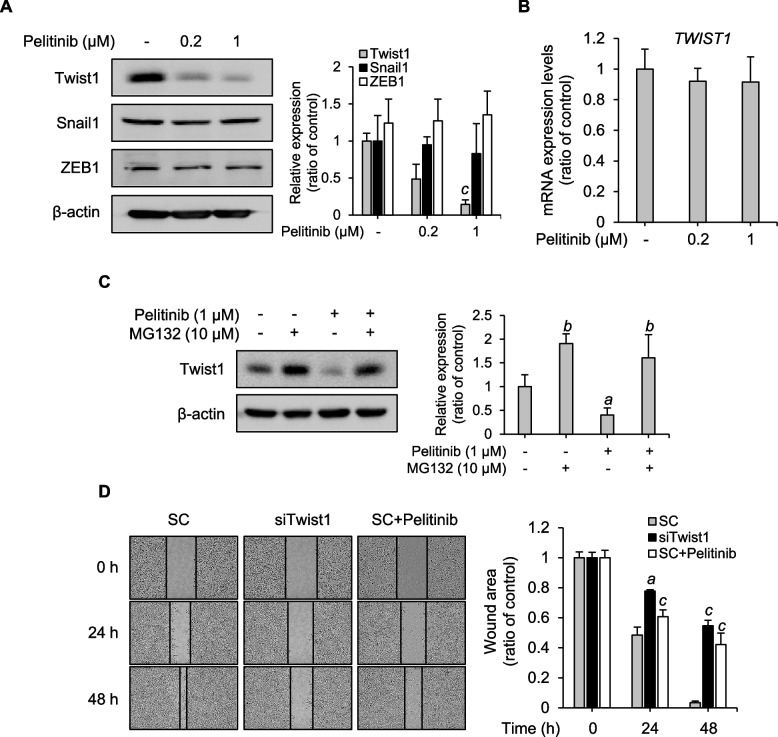
Inhibition of cell migration through regulation of Twist1 protein stability by pelitinib. **A** Huh7 cells were treated with various concentrations of pelitinib for 48 h in media supplemented with 10% FBS. The protein levels of Twist1, Snail1, ZEB1, and β-actin (loading control) were analyzed by immunoblotting. The bar graph shows the quantified protein levels. The data shown are representative of three independent experiments and expressed as the means ± SD. The original gel images are shown in the additional file. The one-way ANOVA is then used to analyze the data. ^c^*p* < 0.001 relative to the pelitinib-untreated group. **B** After pelitinib treatment in Huh7 cells for 48 h, *TWIST1* expression levels were determined by RT-qPCR. The mRNA expression levels of *TWIST1* were normalized by *GAPDH*. The data shown are averages from three independent experiments and the values are expressed as the means ± SD. **C** Prior to 48 h of pelitinib treatment, the cells were pretreated with MG132 (10 μM) for 3 h. The protein levels of Twist1 and β-actin were analyzed by immunoblotting. The bar graph shows quantified protein levels. The data shown are averages of three independent experiments and the values are expressed as the means ± SD. The original gel images are shown in the additional file. The data were analyzed by one-way ANOVA. ^a^*p* < 0.05 and ^b^*p* < 0.01 relative to both pelitinib and MG132 untreated control group. **D** Huh7 cells were transfected with scrambled (SC) siRNA (100 nM) or Twist1-siRNA (100 nM) with Lipofectamine and incubated for 48 h. The wound was introduced using an SPL scratcher, and the cells were subsequently treated with 1 μM of pelitinib in media with 1% FBS. Microscopic images were captured at indicated time points. The wound area was expressed as the percentage of initial wound size for each sample and visualized on a bar graph. The data shown are averages from three independent experiments and expressed as the means ± SD. The one-way ANOVA is then used to analyze the data. ^a^*p* < 0.05 and ^c^*p* < 0.001 relative to the pelitinib-untreated control group

Since EMT-TFs are critical for promoting HCC cell invasion, migration, and metastasis, next we asked whether pelitinib targeted EMT-TFs [43]. To examine whether pelitinib-mediated suppression of migration and invasion of HCC cell lines was due to EMT-TFs, the expression levels of EMT-TFs were analyzed. When Huh7 cells were treated with pelitinib, Twist1 protein levels were reduced in a dose-dependent manner, but no changes in Snail1 and ZEB1 protein levels were detected (Fig. 3A). Moreover, Twist1 protein levels in Hep3B and SNU449 were reduced by pelitinib in a dose-dependent manner (Supplement Fig. 2A and B). As the protein levels of Twist1 were reduced by pelitinib, the suppressive effects of pelitinib on the Twist1 expression were examined at the transcriptional level by RT-qPCR. *TWIST1* mRNA levels were not altered by pelitinib (Fig. 3B), indicating that pelitinib reduced the protein levels but not mRNA levels of Twist1. Accordingly, to investigate the mechanism underlying the regulation of Twist1 protein levels by pelitinib, the proteasomal degradation of Twist1 was evaluated by treating the cells with MG132, a cell-permeable inhibitor of the proteasome. When Huh7 cells were treated with pelitinib, Twist1 protein levels were suppressed as expected, but this suppression was abolished by co-treatment with MG132 (Fig. 3C). These findings imply that the significant reduction of Twist1 protein levels by pelitinib is mediated by proteasomal degradation of Twist1 protein rather than changes in gene expression. Since pelitinib reduces Twist1 protein levels, we investigated various intracellular signaling pathways, such as MAPKs and Akt, that regulate Twist1 protein stability through phosphorylation [27, 44, 45]. Pelitinib treatment induced a significant reduction in the phosphorylation levels of p38, JNK, ERK, and Akt without affecting total protein levels (Supplement Fig. 3A and B). Moreover, Twist1 protein levels by pelitinib and inhibitors of MAPKs and Akt were compared (Supplement Fig. 3C). Comparing Twist1 protein levels to those of JNK (SP600125), p38 (SB203580), ERK (U0126), and Akt (LY294002) inhibitors, pelitinib (1 μM) decreased Twist1 protein levels the most. Taken together, these results suggest that the anti-migration and anti-invasion effects of pelitinib on HCC cell lines are regulated through the inhibition of Twist1.

To confirm the effect of the Twist1 protein expression on cell migration, the wound healing activity was assessed after transfecting Huh7 cells with Twist1-siRNA. We confirmed that endogenous Twist1 protein levels were decreased in Twist1-siRNA transfected Huh7 cells when compared to non-transfected cells (Supplement Fig. 4). Wound closure by Twist1-siRNA transfected cells was suppressed compared to the control group (Fig. 3D). Moreover, Huh7 cells transfected with Twist1-siRNA exhibited wound closure inhibition at levels comparable those in pelitinib-treated Huh7 cells. These results confirm that the inhibition of Twist1 is associated with the suppression of migration in Huh7 cells and suggest that the suppression of wound closure by pelitinib treatment is due to a reduction in Twist1 levels.

### Pelitinib regulates the expression of E-cadherin and N-cadherin, the main target genes of Twist1

**Fig. 4:**
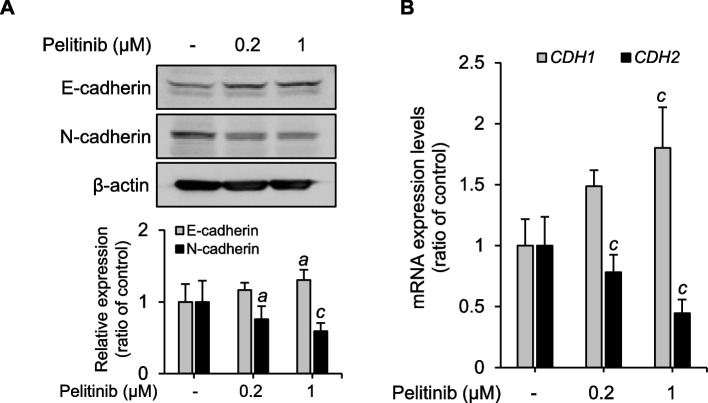
Regulation of E-cadherin and N-cadherin expression by pelitinib. **A** Huh7 cells were treated with various concentrations of pelitinib for 48 h. Protein levels of E-cadherin, N-cadherin, and β-actin (loading control) were determined by immunoblotting. The quantitative analysis of the intensity of the bands on the immunoblot is shown in the bar graph. The data shown are representative of three experiments and expressed as the means ± SD. The original gel images are shown in the additional file. These data are subjected to one-way ANOVA. ^a^*p* < 0.05 and ^c^*p* < 0.001 relative to the pelitinib-untreated control group. **B** *CDH1* and *CDH2* mRNA levels in Huh7 cells were determined by RT-qPCR. Huh7 cells were treated with pelitinib at each concentration for 48 h. *GAPDH* was utilized for normalization. In both panels, the data shown are averages from three independent experiments and expressed as the means ± SD. The one-way ANOVA is then used to analyze the data. ^c^*p* < 0.001 relative to the pelitinib-untreated control group

As the changes in E-cadherin and N-cadherin levels are the hallmarks of EMT, the changes in the expression levels of these two main EMT target genes were examined [46]. In parallel to the decreases in Twist1 levels upon pelitinib treatment, protein levels of E-cadherin were elevated, while those of N-cadherin protein were reduced; both changes were statistically significant (Fig. 4A). E-cadherin (*CDH1*) and N-cadherin (*CDH2*) mRNA levels, as measured by RT-qPCR, mirrored the changes noted in the protein levels (Fig. 4B). Taken together, these results imply that the suppression of Twist1 protein levels by pelitinib alters E-cadherin and N-cadherin expression, which may prevent further cancer progression.

## Discussion

Metastasis remains the leading cause of cancer-related death despite therapeutic strategies for HCC, and although FDA-approved drugs are available, the survival rate of patients with metastatic HCC is still low [47]. Therefore, it is crucial to identify efficacious therapeutic inhibitors of cancer migration and metastasis with potential clinical applications. Herein, this study reveals that pelitinib exerts anti-migration and anti-invasion activities in HCC via inhibition of Twist1 signaling pathways.

Among the EMT-TFs, the overexpression of Twist1 is linked to poor prognosis in patients with various cancer types [48–51]. Moreover, Twist1 expression is upregulated in patients receiving neoadjuvant chemotherapy, which is correlated with chemoresistance [50]. Twist1 is stabilized by several EMT-related signaling pathways, such as Akt and MAPKs [52, 53]. These signaling pathways are activated by phosphorylation of EGFR at the tyrosine domain [54, 55]. A previous study related to pelitinib demonstrated that pelitinib suppressed the activation of Akt and ERK and led to suppression of proliferation [37]. In Huh7 cells with high EGFR expression [56], pelitinib was found to decrease phosphorylation of p38 and JNK as well as Akt and ERK. In comparison to a previous study related to pelitinib, the present study uses a lower dose of pelitinib to inhibit Akt and MAPK signaling pathways [37]. Moreover, this study showed that pelitinib significantly downregulated Twist1 protein levels in a dose-dependent manner in Huh7 cells, but the mRNA levels were unchanged. In addition, the protein levels of two other EMT-TFs, Snail1 and ZEB1, were not altered by pelitinib, suggesting that pelitinib specifically targets Twist1. Although pelitinib treatment inhibits only Twist1 protein levels, it results in a dramatic suppression of migration and invasion in Huh7 cells. This observation encouraged us to further investigate how the protein levels of Twist1 were regulated by pelitinib. In Huh7 cells, the pelitinib-mediated reduction in Twist1 protein levels was reversed upon co-treatment with MG132, a proteasome inhibitor. These results suggest that pelitinib treatment induces the degradation of Twist1 through ubiquitin-mediated proteolysis in Huh7 cells, indicating that pelitinib functions as a new inhibitor that regulates Twist1 at the post-translational level. The results indicate that pelitinib has inhibitory effects on both Akt and MAPKs, leading to Twist1 inhibition in Huh7 cells. Comparing treatments with the same or greater concentrations of Akt and MAPK inhibitors, it is interesting to note that pelitinib demonstrated the greatest Twist1 reduction. As a result, Twist1 protein levels were significantly inhibited by pelitinib. This is, to our knowledge, the first study to examine how pelitinib inhibits Twist1-mediated cell invasion and migration in HCC cell lines.

The targeting of Twist1 but not the other EMT-TFs by pelitinib might be explained by a variety of reasons. A study by Lee et al. has shown that RNF8, an E3 ligase, acts as a Twist1 activator via ubiquitination [57]. It is possible that pelitinib may directly or indirectly target RNF8, leading to the decrease in Twist1 stability. In addition, Twist1 is degraded by other E3 ligases, including Pirh2 and tumor necrosis factor receptor-associated factor-interacting protein [58, 59]. Considering the findings reported to date, these E3 ligases target only Twist1 and induce its degradation by ubiquitination. Pelitinib may cause degradation of Twist1 by activating these E3 ligases through unidentified pathways. Twist1-specific E3 ligase activity may be one of the factors contributing to the reduction in Twist1 protein stability by pelitinib, despite the fact that the detailed molecular mechanisms of the aforementioned E3 ligase have not been thoroughly investigated. The other possibility is that, even though Akt and MAPK signaling pathways stabilize Snail1, there could be other signaling pathways involved to maintain the stability of Snail1 in pelitinib-treated cells [60, 61]. The regulatory mechanisms of ZEB1 stability in relation to Akt and MAPKs are not well-studied yet. Due to these factors, further research is necessary for the identification of the molecular mechanisms by which pelitinib targets Twist1.

Clinical samples of HCC have shown that low expression of E-cadherin mRNA in metastatic cell lines is correlated with high expression of Twist1 [62]. N-cadherin expression, another hallmark of EMT, is reduced in Twist1 knockdown NSCLC cells, leading to the inhibition of apoptosis and invasive behavior in lung cancer cells [63]. The present study demonstrated that pelitinib upregulates mRNA and protein levels of E-cadherin while downregulating those of N-cadherin, suggesting that pelitinib effectively inhibits EMT in Huh7 cells. Furthermore, the overexpression of MMPs, especially MMP-2 and -9, in the cancer microenvironment facilitates EMT via architectural changes in cells and tissues [64–67]. A study by Khales et al. has shown that overexpression of Twist1 in cells increases the activities of MMP-2 and -9 [68]. In addition, activities of MMP-2 and -9 in HCC have been shown to be associated with an increase in metastasis after resection [69]. Our data showed that pelitinib dose-dependently reduced activities of endogenous MMP-2 and -9 in Huh7 cells. In addition to the regulation of E-cadherin and N-cadherin in EMT, the reductions in MMP-2 and -9 levels could be additional mediators of pelitinib action that could lead to the suppression of EMT-mediated invasion and migration in HCC. Overall, our results indicate that the regulatory effect of pelitinib on Twist1 is closely associated with the alterations in the expression levels of EMT-related genes and in the migratory and invasive properties of HCC.

However, it is important to note that the experiments conducted in this study were limited to in vitro cellular assays, and further in vivo studies are required to elucidate the direct mechanism of action of pelitinib in inhibiting HCC metastasis. If pelitinib is shown to be effective in vivo as well, it may offer a promising therapeutic strategy for the treatment of HCC. In addition, considering the approval of other tyrosine kinase inhibitors for the treatment of various cancer types, as well as the FDA-approved drugs for HCC [70–72], precision combination therapy using pelitinib along with these inhibitors or drugs may have synergistic effects in the treatment of HCC, which is a promising area of research for future studies.

## Conclusions

This study demonstrates the anti-migration and anti-invasion effects of pelitinib and the underlying molecular mechanism in Huh7 cells. The inhibition of the Akt- and MAPKs-mediated Twist1 signaling pathways by pelitinib resulted in a dramatic suppression of EMT-associated cellular functions, including cell migration, invasion, and MMP-2 and -9 activities. Hence, understanding the actions of pelitinib on Twist1 through Akt and MAPK signaling pathways may provide the basis for potential anti-cancer metastatic therapies and could help improve the survival rate of patients with HCC.

## Supplementary Information

**Additional file 1: Supplementary Figure 1.** Suppressive effects of pelitinib on cell migration and viability in Hep3B and SNU449. **Supplementary Figure 2.** Inhibition of pelitinib on Twist1 protein levels in Hep3B and SNU449. **Supplementary Figure 3.** Inhibitory effects of pelitinib on the activation of Akt and MAPK signaling pathways. **Supplementary Figure 4.** Confirmation of reduced Twist1 protein levels in Twist1-siRNA transfected cells.

**Additional file 2. **Original uncropped gel images.

## Data Availability

The original gel images supporting the conclusions of this article are included within its additional file.

## Abbreviations

MAPK: Mitogen-activated protein kinase
HCC: Hepatocellular carcinoma
EMT: Epithelial-mesenchymal transition
EMT-TF: Epithelial-mesenchymal transition-transcription factor
ECM: Extracellular matrix
MMP: Matrix metalloproteinase
NSCLC: Non-small cell lung cancer
ERK: Extracellular signal-regulated kinase
JNK: C-Jun N-terminal Kinase
FDA: Food and drug administration
EGFR: Epidermal growth factor receptor
GAPDH: Glyceraldehyde 3-phosphate dehydrogenase
PBS: Phosphate-buffered saline
TBST: Tris‑buffered saline with Tween 20
SDS: Sodium dodecyl sulfate
SD: Standard deviation
FBS: Fetal bovine serum
RT-qPCR: Quantitative reverse transcription polymerase chain reaction
EGFR-TK: Epidermal growth factor receptor tyrosine kinase
