# Supplementary material for: Role of pelitinib in the regulation of migration and invasion of hepatocellular carcinoma cells via inhibition of Twist1
Source: BMC Cancer. 2023 Jul 27;23:703. doi: 10.1186/s12885-023-11217-2 (PMC10373356; doi:10.1186/s12885-023-11217-2)
Supplement: Supplementary file 1 — Additional file 1: Supplementary Figure 1. Suppressive effects of pelitinib on cell migration and viability in Hep3B and SNU449. Supplementary Figure 2. Inhibition of pelitinib on Twist1 protein levels in Hep3B and SNU449. Supplementary Figure 3. Inhibitory effects of pelitinib on the activation of Akt and MAPK signaling pathways. Supplementary Figure 4. Confirmation of reduced Twist1 protein levels in Twist1-siRNA transfected cells. [file 12885_2023_11217_MOESM1_ESM.docx]

Supplementary Materials for

(This file contains supplementary figures S1–S4)

Role of pelitinib in the regulation of migration and invasion of hepatocellular carcinoma cells via inhibition of Twist1

**Sewoong Lee**†**, Eunjeong Kang**†, **Unju Lee, Sayeon Cho^*^**

† These authors contributed equally to this work.

Laboratory of Molecular and Pharmacological Cell Biology, College of Pharmacy, Chung-Ang University, Seoul 06974, Republic of Korea

Sewoong Lee: dltpdnd2000@naver.com, Eunjeong Kang: ejaykang@gmail.com, Unju Lee: unjulee98@gmail.com, Sayeon Cho: sycho@cau.ac.kr

***** Correspondence to: Sayeon Cho, E-mail: [sycho@cau.ac.kr](mailto:sycho@cau.ac.kr)


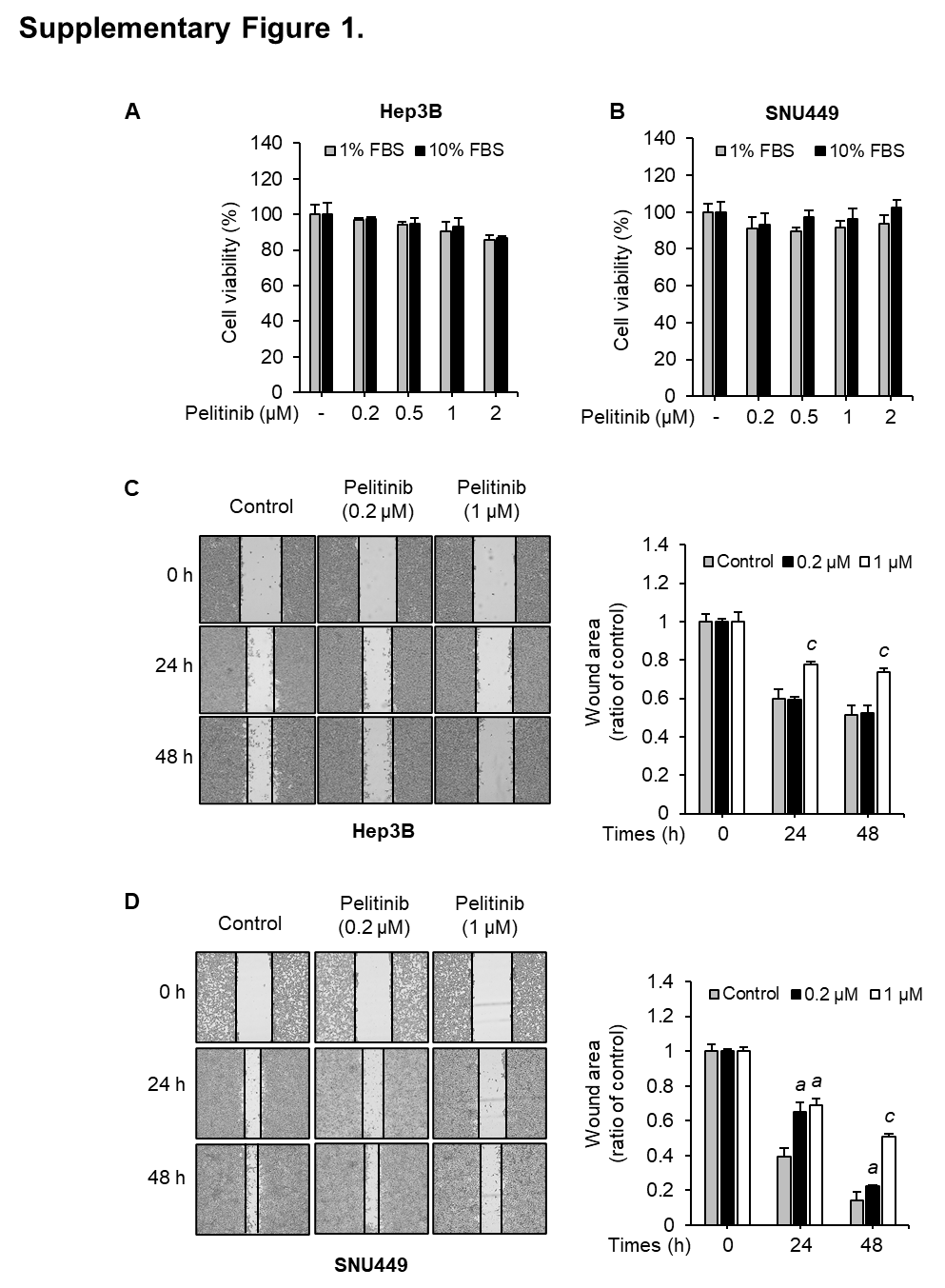


**Supplementary Figure 1. Suppressive effects of pelitinib on cell migration and viability in Hep3B and SNU449.** (A) Hep3B and (B) SNU449 cells were treated with different concentrations of pelitinib for 48 h. Cell viability assay was tested in cell culture media containing 1% or 10% FBS and determined using EZ-Cytox kit. The cell viability was expressed relative to the untreated control group and visualized on a bar graph. Wound-healing assays in (C) Hep3B and (D) SNU449 cells were performed with pelitinib for 48 h. After cell seeding, a plastic SPL scratcher was used to make a wound in plates, and then cells were incubated with pelitinib in 1% FBS-containing media. The microscopic pictures were captured at indicated time points. By calculating the percentage of wound closure relative to the 0 h point for each sample, the wound closure values were quantified. The relative wound closure was shown as a bar graph. The data shown are averages from three independent experiments and expressed as the means ± SD. The one-way ANOVA is then used to analyze the data. ^a^*p* < 0.05 and ^c^*p* < 0.001 relative to the pelitinib-untreated control group.


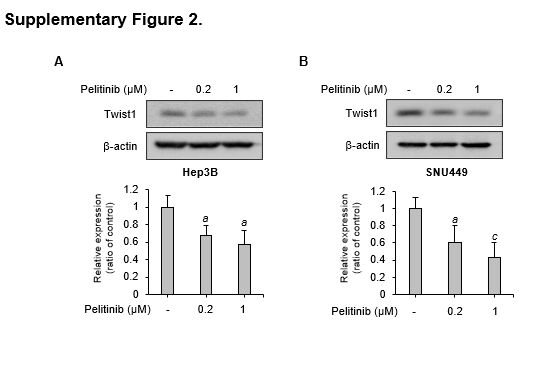


**Supplementary Figure 2. Inhibition of pelitinib on Twist1 protein levels in Hep3B and SNU449.** (A) Hep3B and (B) SNU449 cells were treated with pelitinib for 48 h in media with 10% FBS. The protein levels of Twist1 and β-actin (loading control) were analyzed by immunoblotting. The quantitative analysis of the intensity of the bands on the immunoblot is shown in the bar graph. The original gel images are shown in the additional file. The data shown are representative of three experiments and expressed as the means ± SD and analyzed by one-way ANOVA. ^a^*p* < 0.05 and ^c^*p* < 0.001 relative to the pelitinib-untreated control group.


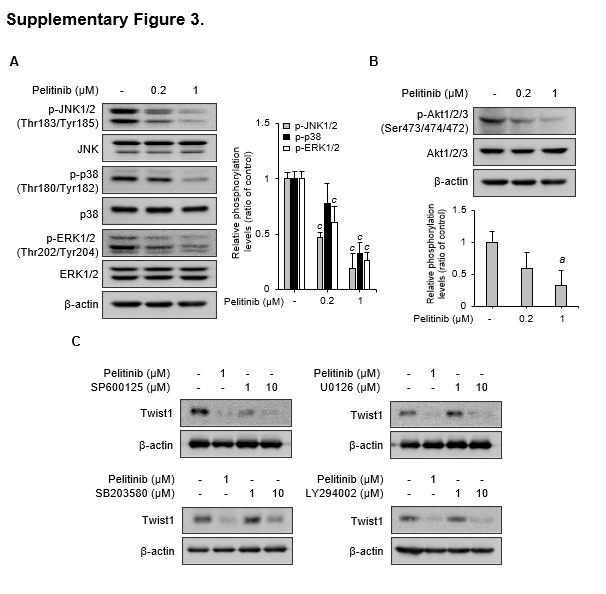


**Supplementary Figure 3. Inhibitory effects of pelitinib on the activation of Akt and MAPK signaling pathways.** (A and B) Huh7 cells were treated with pelitinib for 48 h. Expression levels of (A) MAPK and (B) Akt signaling pathway-related proteins were analyzed by immunoblotting. As a loading control, β-actin was used. The phosphorylation levels of protein were normalized by total protein levels. The bar graph shows quantified protein levels. The data are expressed as means ± SD of three independent experiments. The one-way ANOVA is then used to analyze the data. ^a^*p* < 0.05 and ^c^*p* < 0.001 relative to the pelitinib-untreated control group. (C) Huh7 cells were treated with pelitinib or inhibitors of JNK (SP600125), p38 (SB203580), ERK (U0126), and Akt (LY294002) for 48 h. Twist1 protein levels were analyzed by immunoblotting. As a loading control, β-actin was used. All the original gel images are shown in the additional file.


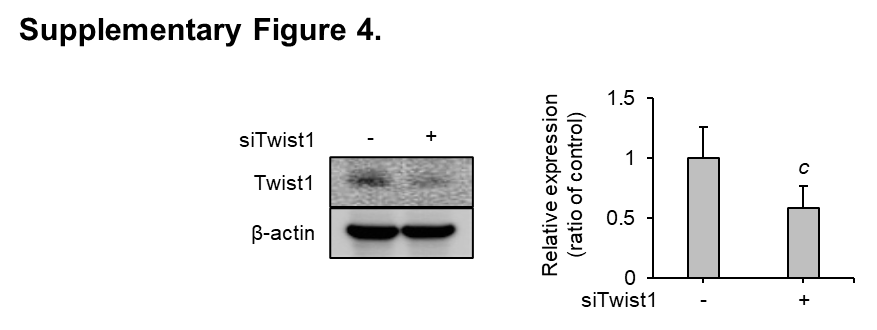


**Supplementary Figure 4. Confirmation of reduced Twist1 protein levels in Twist1-siRNA transfected cells.** Using Lipofectamine, Huh7 cells were transfected with scrambled siRNA (100 nM) or Twist1-siRNA (100 nM) and incubated at 37 °C for 48 h. Inhibition of Twist1 expression by siRNA was confirmed by immunoblotting. The bar graph shows quantified protein levels. The original gel images are shown in the additional file. The data shown are the averages from three independent experiments and expressed as the means ± SD. The one-way ANOVA is then used to analyze the data. ^c^*p* < 0.001 relative to the pelitinib-untreated control group.
