# Supplementary material for: Role of pelitinib in the regulation of migration and invasion of hepatocellular carcinoma cells via inhibition of Twist1
Source: BMC Cancer. 2023 Jul 27;23:703. doi: 10.1186/s12885-023-11217-2 (PMC10373356; doi:10.1186/s12885-023-11217-2)
Supplement: Supplementary file 2 — Additional file 2. Original uncropped gel images. [file 12885_2023_11217_MOESM2_ESM.pptx]

## Slide 1
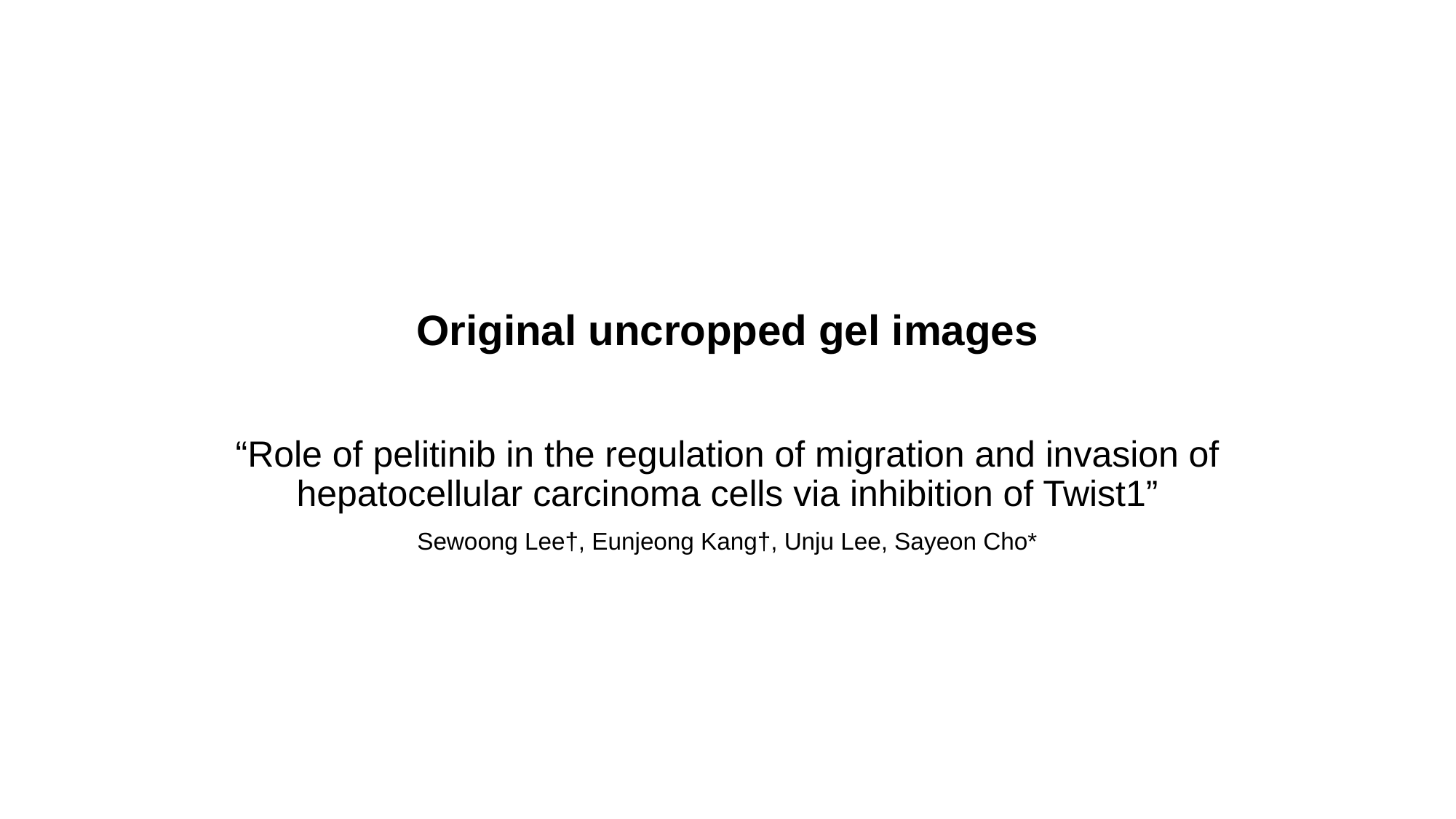

# Original uncropped gel images
“Role of pelitinib in the regulation of migration and invasion of hepatocellular carcinoma cells via inhibition of Twist1”
Sewoong Lee†, Eunjeong Kang†, Unju Lee, Sayeon Cho*

## Slide 2
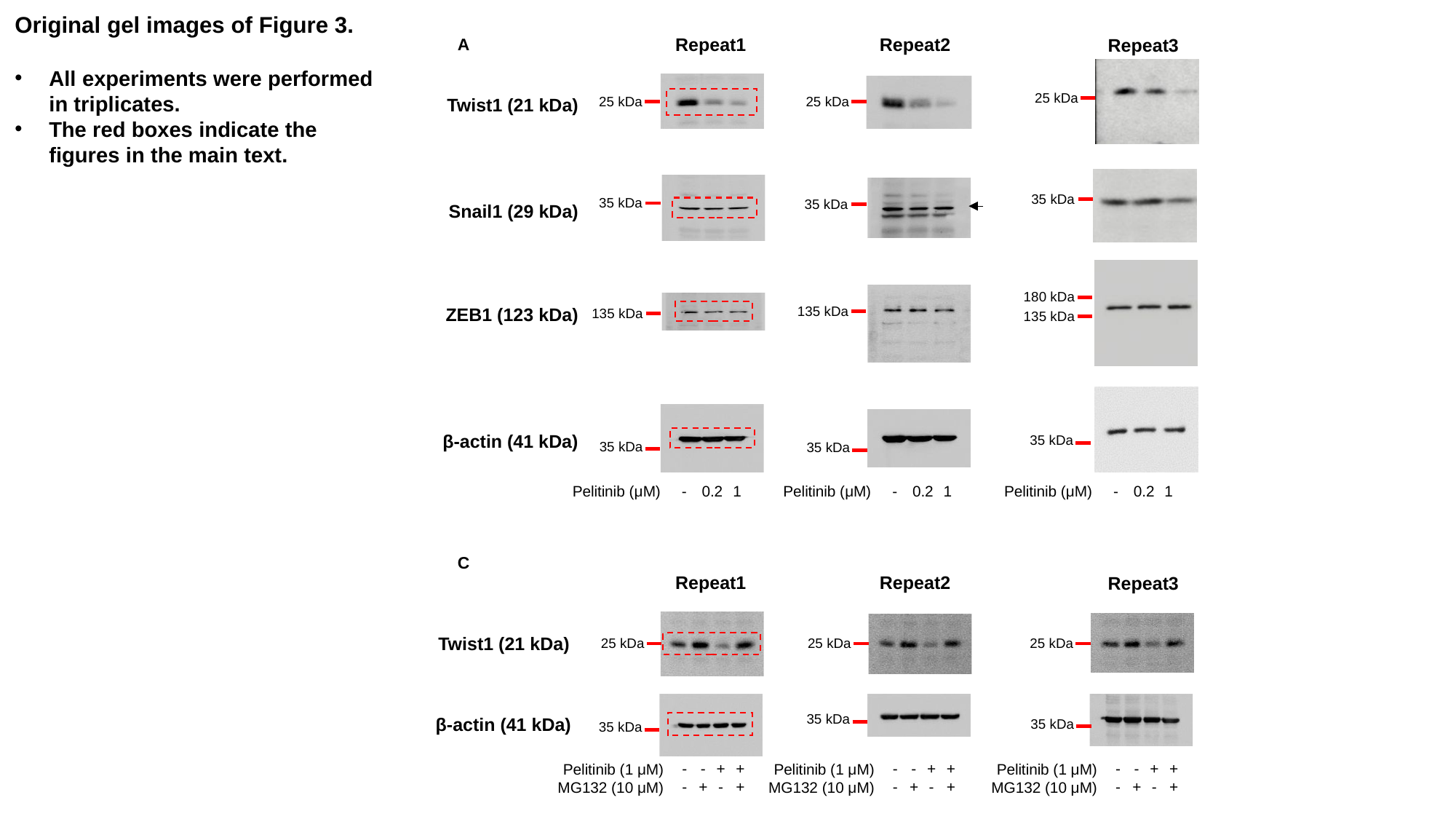

Original gel images of Figure 3.
All experiments were performed in triplicates.
The red boxes indicate the figures in the main text.
Repeat1
Repeat2
Repeat3
A
25 kDa
25 kDa
25 kDa
Twist1 (21 kDa)
35 kDa
35 kDa
35 kDa
Snail1 (29 kDa)
180 kDa
135 kDa
ZEB1 (123 kDa)
135 kDa
135 kDa
β-actin (41 kDa)
35 kDa
35 kDa
35 kDa
Pelitinib (μM)
-
0.2
1
Pelitinib (μM)
-
0.2
1
Pelitinib (μM)
-
0.2
1
C
Repeat1
Repeat2
Repeat3
Twist1 (21 kDa)
25 kDa
25 kDa
25 kDa
35 kDa
β-actin (41 kDa)
35 kDa
35 kDa
-
-
+
+
-
-
+
+
-
-
+
+
-
+
-
+
-
+
+
-
+
-
+
-
Pelitinib (1 μM)
MG132 (10 μM)
Pelitinib (1 μM)
MG132 (10 μM)
Pelitinib (1 μM)
MG132 (10 μM)

## Slide 3
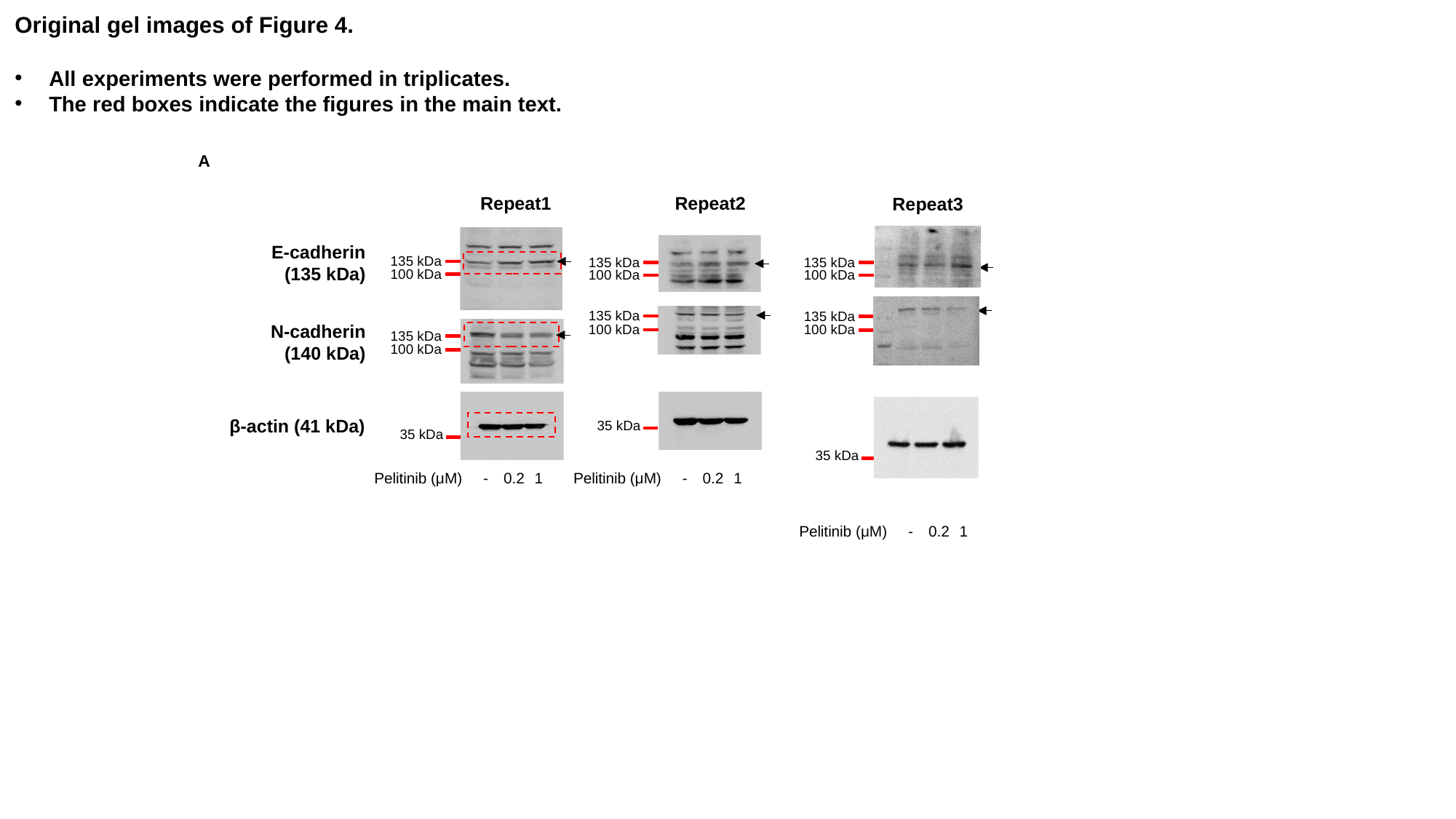

Original gel images of Figure 4.
All experiments were performed in triplicates.
The red boxes indicate the figures in the main text.
A
Repeat1
Repeat2
Repeat3
E-cadherin (135 kDa)
135 kDa
135 kDa
135 kDa
100 kDa
100 kDa
100 kDa
135 kDa
135 kDa
N-cadherin (140 kDa)
100 kDa
100 kDa
135 kDa
100 kDa
β-actin (41 kDa)
35 kDa
35 kDa
35 kDa
Pelitinib (μM)
-
0.2
1
Pelitinib (μM)
-
0.2
1
Pelitinib (μM)
-
0.2
1

## Slide 4
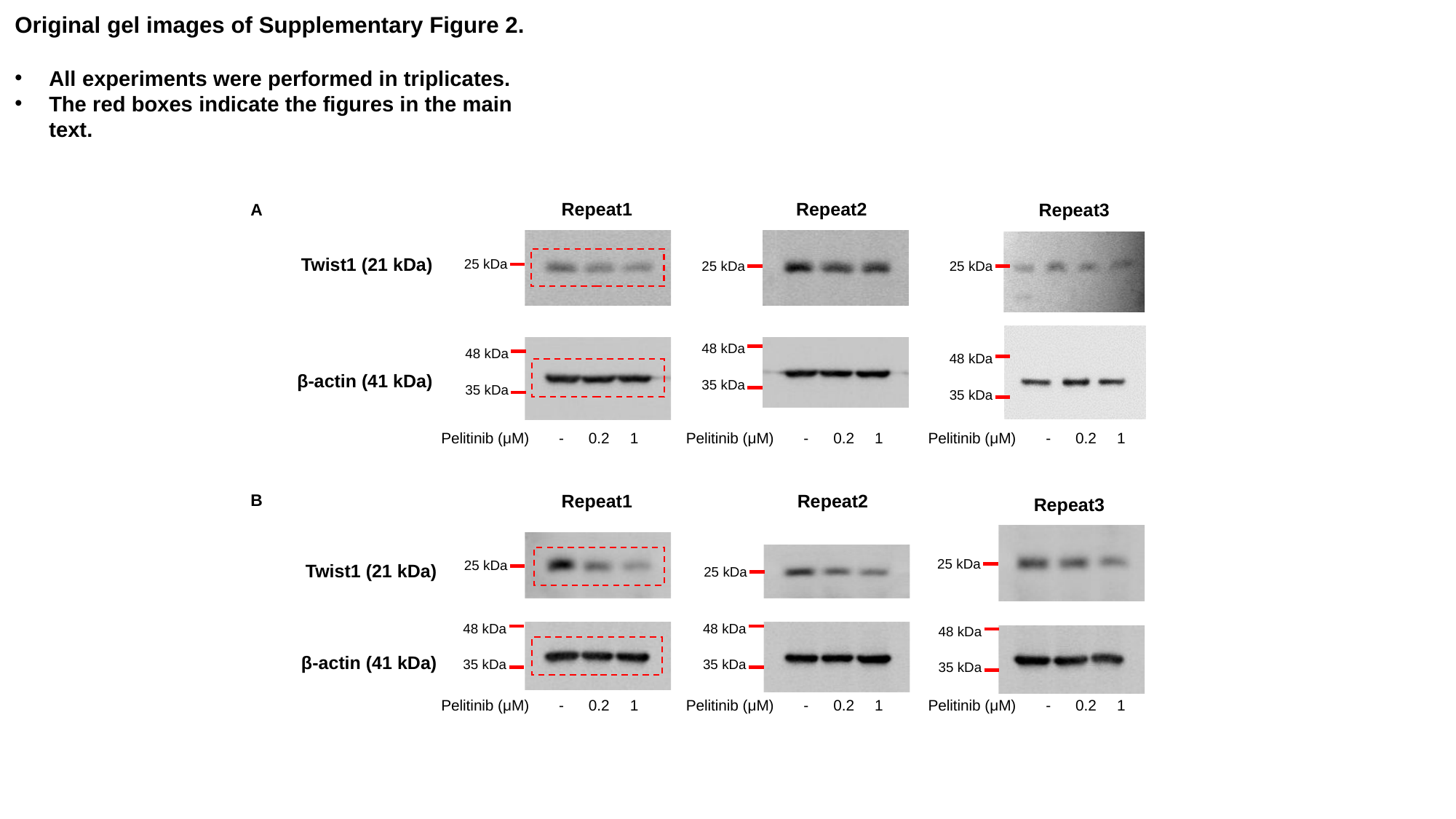

Original gel images of Supplementary Figure 2.
All experiments were performed in triplicates.
The red boxes indicate the figures in the main text.
Repeat1
Repeat2
Repeat3
A
Twist1 (21 kDa)
25 kDa
25 kDa
25 kDa
48 kDa
48 kDa
48 kDa
β-actin (41 kDa)
35 kDa
35 kDa
35 kDa
Pelitinib (μM)
-
0.2
1
Pelitinib (μM)
-
0.2
1
Pelitinib (μM)
-
0.2
1
Repeat1
Repeat2
B
Repeat3
25 kDa
25 kDa
Twist1 (21 kDa)
25 kDa
48 kDa
48 kDa
48 kDa
β-actin (41 kDa)
35 kDa
35 kDa
35 kDa
Pelitinib (μM)
-
0.2
1
Pelitinib (μM)
-
0.2
1
Pelitinib (μM)
-
0.2
1

## Slide 5
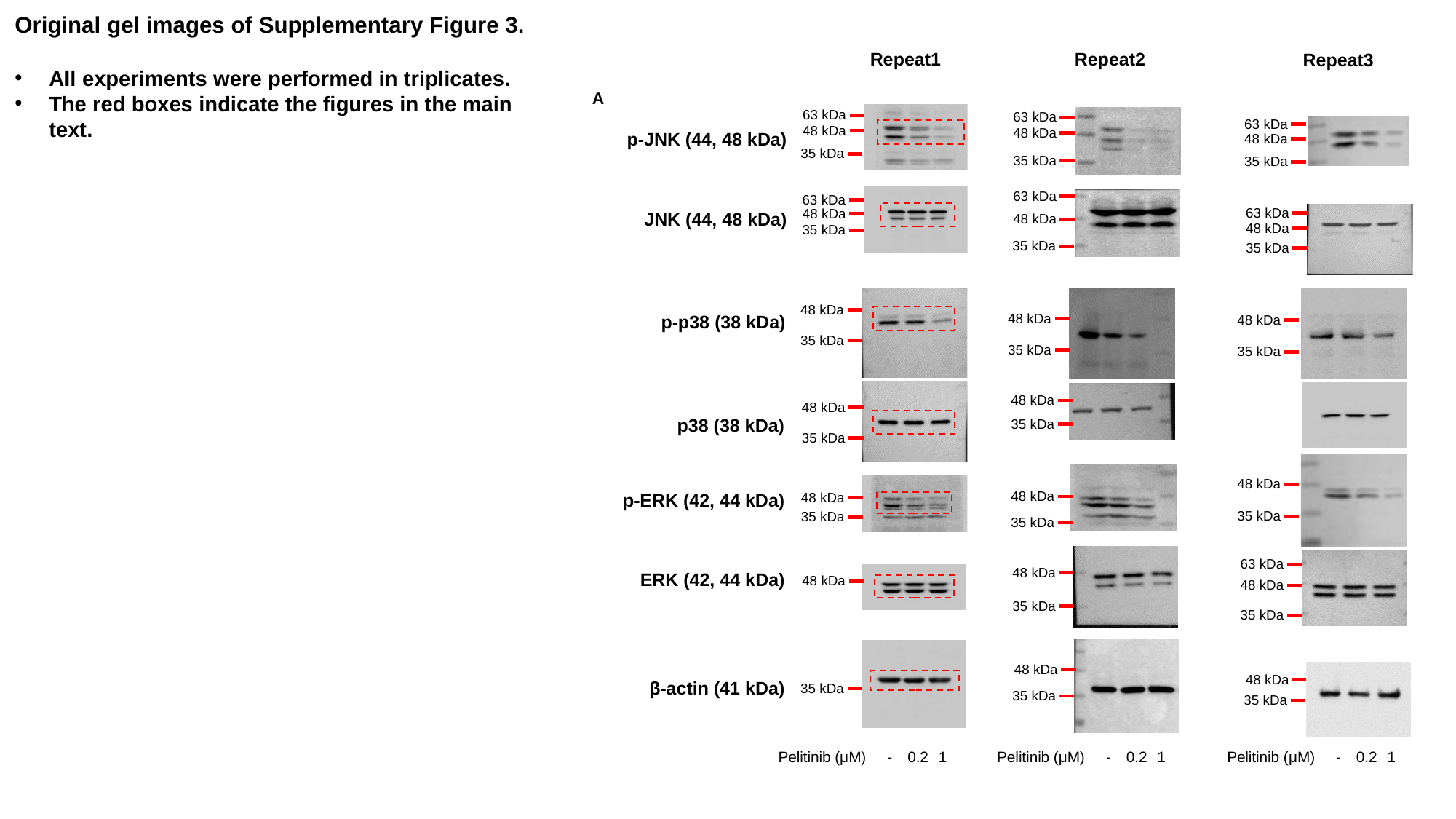

Original gel images of Supplementary Figure 3.
All experiments were performed in triplicates.
The red boxes indicate the figures in the main text.
Repeat1
Repeat2
Repeat3
A
63 kDa
63 kDa
63 kDa
48 kDa
48 kDa
p-JNK (44, 48 kDa)
48 kDa
35 kDa
35 kDa
35 kDa
63 kDa
63 kDa
63 kDa
48 kDa
JNK (44, 48 kDa)
48 kDa
48 kDa
35 kDa
35 kDa
35 kDa
48 kDa
48 kDa
p-p38 (38 kDa)
48 kDa
35 kDa
35 kDa
35 kDa
48 kDa
48 kDa
p38 (38 kDa)
35 kDa
35 kDa
48 kDa
48 kDa
p-ERK (42, 44 kDa)
48 kDa
35 kDa
35 kDa
35 kDa
63 kDa
48 kDa
ERK (42, 44 kDa)
48 kDa
48 kDa
35 kDa
35 kDa
48 kDa
48 kDa
β-actin (41 kDa)
35 kDa
35 kDa
35 kDa
Pelitinib (μM)
-
0.2
1
Pelitinib (μM)
-
0.2
1
Pelitinib (μM)
-
0.2
1

## Slide 6
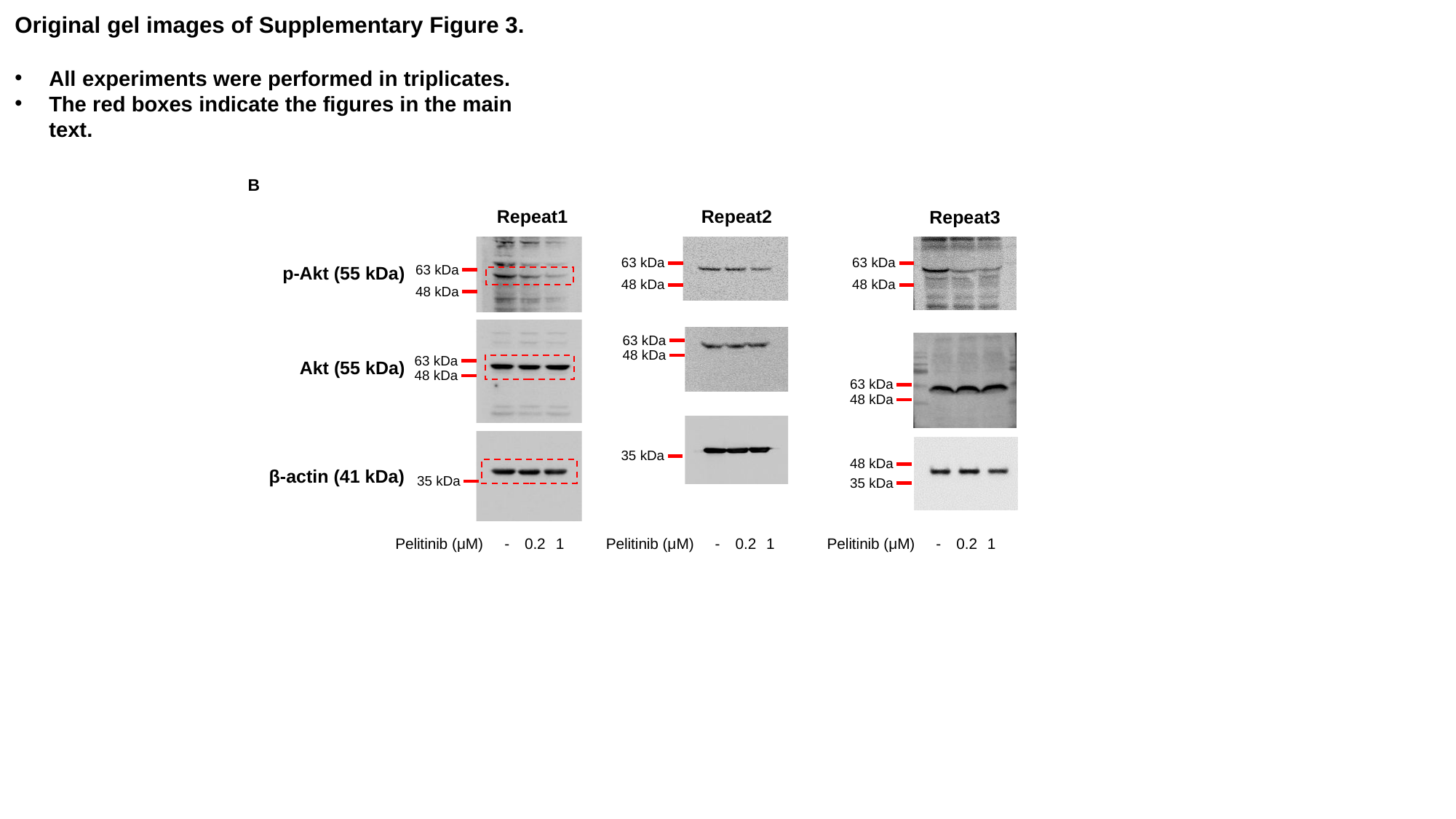

Original gel images of Supplementary Figure 3.
All experiments were performed in triplicates.
The red boxes indicate the figures in the main text.
B
Repeat1
Repeat2
Repeat3
63 kDa
63 kDa
63 kDa
p-Akt (55 kDa)
48 kDa
48 kDa
48 kDa
63 kDa
48 kDa
63 kDa
Akt (55 kDa)
48 kDa
63 kDa
48 kDa
35 kDa
48 kDa
β-actin (41 kDa)
35 kDa
35 kDa
Pelitinib (μM)
-
0.2
1
Pelitinib (μM)
-
0.2
1
Pelitinib (μM)
-
0.2
1

## Slide 7
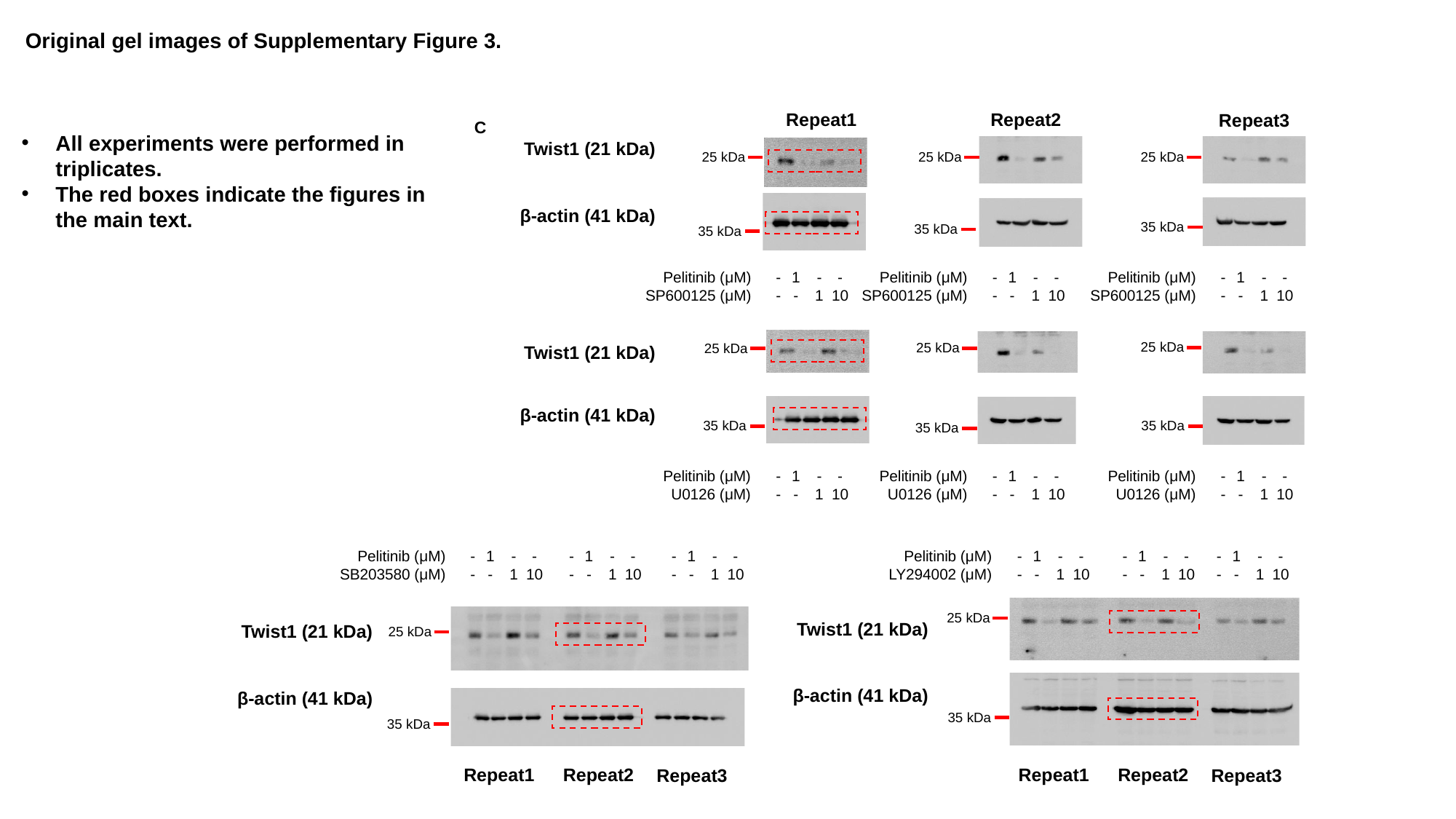

Original gel images of Supplementary Figure 3.
All experiments were performed in triplicates.
The red boxes indicate the figures in the main text.
Repeat1
Repeat2
Repeat3
C
Twist1 (21 kDa)
25 kDa
25 kDa
25 kDa
β-actin (41 kDa)
35 kDa
35 kDa
35 kDa
Pelitinib (μM)
SP600125 (μM)
-
-
1
-
-
1
-
10
Pelitinib (μM)
SP600125 (μM)
-
-
1
-
-
1
-
10
Pelitinib (μM)
SP600125 (μM)
-
-
1
-
-
1
-
10
25 kDa
25 kDa
25 kDa
Twist1 (21 kDa)
β-actin (41 kDa)
35 kDa
35 kDa
35 kDa
Pelitinib (μM)
U0126 (μM)
-
-
1
-
-
1
-
10
Pelitinib (μM)
U0126 (μM)
-
-
1
-
-
1
-
10
Pelitinib (μM)
U0126 (μM)
-
-
1
-
-
1
-
10
Pelitinib (μM)
SB203580 (μM)
-
-
1
-
-
1
-
10
-
-
1
-
-
1
-
10
-
-
1
-
-
1
-
10
Pelitinib (μM)
LY294002 (μM)
-
-
1
-
-
1
-
10
-
-
1
-
-
1
-
10
-
-
1
-
-
1
-
10
25 kDa
Twist1 (21 kDa)
Twist1 (21 kDa)
25 kDa
β-actin (41 kDa)
β-actin (41 kDa)
35 kDa
35 kDa
Repeat1
Repeat2
Repeat1
Repeat2
Repeat3
Repeat3

## Slide 8
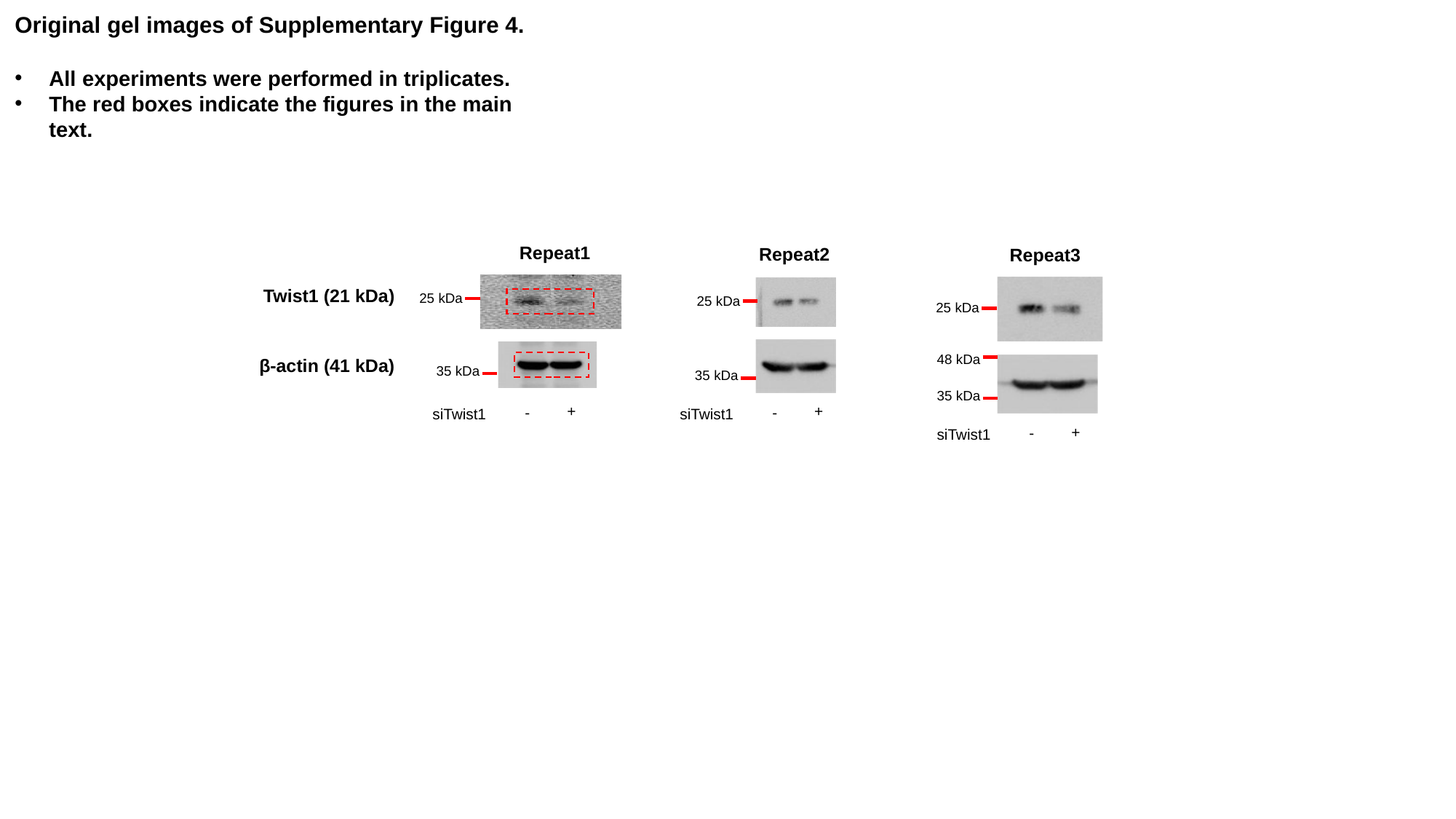

Original gel images of Supplementary Figure 4.
All experiments were performed in triplicates.
The red boxes indicate the figures in the main text.
Repeat1
Repeat2
Repeat3
Twist1 (21 kDa)
25 kDa
25 kDa
25 kDa
48 kDa
β-actin (41 kDa)
35 kDa
35 kDa
35 kDa
+
+
-
-
siTwist1
siTwist1
+
-
siTwist1
